# Poly(Lactic-Co-Glycolic Acid)-Based Systems in Implantology: Advances in Biomaterial Design, Drug Delivery, and Tissue Regeneration

**DOI:** 10.3390/polym18091113

**Published:** 2026-04-30

**Authors:** Bogdan Alexandru Popescu, Ionela Belu, Andreea Gabriela Mocanu, Maria Viorica Ciocîlteu, Daniela Calina, Costel Valentin Manda, Johny Neamțu, Oana Elena Nicolaescu, Andreea-Cristina Stoian, Andreea Silvia Pîrvu

**Affiliations:** 1 Doctoral School, University of Medicine and Pharmacy of Craiova, 2 Petru Rareş Street, 200349 Craiova, Romania; bogdan.popescu@umfcv.ro; 2 Department of Pharmaceutical Technique, Faculty of Pharmacy, University of Medicine and Pharmacy of Craiova, 2 Petru Rareş Street, 200349 Craiova, Romania; ionela.belu@umfcv.ro (I.B.); oana.nicolaescu@umfcv.ro (O.E.N.); 3 Department of Instrumental and Analytical Chemistry, Faculty of Pharmacy, University of Medicine and Pharmacy of Craiova, 2 Petru Rareş Street, 200349 Craiova, Romania; maria.ciocilteu@umfcv.ro; 4 Department of Clinical Pharmacy, Faculty of Pharmacy, University of Medicine and Pharmacy of Craiova, 2 Petru Rareş Street, 200349 Craiova, Romania; daniela.calina@umfcv.ro; 5 Department of Physics, Faculty of Pharmacy, University of Medicine and Pharmacy of Craiova, 2 Petru Rareş Street, 200349 Craiova, Romania; johny.neamtu@umfcv.ro; 6 Department of Infectious Diseases, Faculty of Medicine, University of Medicine and Pharmacy of Craiova, 2 Petru Rareş Street, 200349 Craiova, Romania; andreea.stoian@umfcv.ro; 7 Department of Biochemistry, Faculty of Medicine, University of Medicine and Pharmacy of Craiova, 2 Petru Rareş Street, 200349 Craiova, Romania; andreea.pirvu@umfcv.ro

**Keywords:** PLGA, implantology, biodegradable polymers, drug delivery, bone regeneration, scaffold design, tissue engineering

## Abstract

Poly(lactic-co-glycolic acid) (PLGA) is one of the most extensively investigated biodegradable polymers for biomedical applications, owing to its tunable degradation kinetics, established biocompatibility, and regulatory approval. In implantology, PLGA-based systems have emerged as versatile platforms for scaffolds, coatings, and localized drug delivery, aimed at enhancing osseointegration and tissue regeneration. This review provides a focused and up-to-date analysis of PLGA applications in dental and orthopedic implantology, with particular emphasis on advances reported over the past decade. Unlike previous reviews that predominantly address general drug delivery or broad tissue engineering applications, this work establishes a direct correlation between polymer composition (LA:GA ratio), processing strategies, and biological outcomes, including degradation behavior, mechanical performance, and host response. Special attention is given to multifunctional PLGA systems incorporating antibiotics, growth factors, and bioactive nanoparticles, highlighting their role in improving antibacterial efficacy and osteogenesis. Emerging technologies such as nanostructured composites, additive manufacturing, and stimuli-responsive delivery platforms are critically evaluated. Key limitations—including acidic degradation by-products, burst release kinetics, and translational barriers—are discussed in the context of clinical applicability. By integrating physicochemical design with biological performance and recent clinical trends (2024–2025), this review proposes a framework for the rational development of next-generation PLGA-based implant systems.

## 1. Introduction

Over the past several decades, implantology has undergone substantial advancements, driven by the need to restore both function and esthetics in dental and orthopedic applications. Conventional metallic implants, including titanium (Ti) alloys, stainless steel, and cobalt–chromium systems, have long been considered the gold standard due to their excellent mechanical strength and durability. However, despite their widespread clinical success, these materials present several limitations that can compromise long-term outcomes [1,2,3,4,5,6,7].

Corrosion and ion release from metallic implants remain a significant concern. In physiological environments, metals can undergo electrochemical corrosion, releasing metal ions (e.g., Ni, Cr, Co) that can induce inflammatory responses, systemic toxicity and reduced bioactivity, ultimately impairing osseointegration [1,2,8]. For example, cobalt–chromium and nickel alloys have demonstrated risks associated with chronic ion exposure [9].

Another limitation is the relative bioinertness of many metallic implants. While titanium and its alloys are relatively well tolerated and can osseointegrate to some extent, many metals do not actively support bone bonding or osteoinduction, often being surrounded by fibrous tissue rather than bone. This gap in functionality can reduce implant stability, result in slow healing, or lead to failure [1,10,11,12].

Mechanical mismatch between implants and native bone tissue further contributes to clinical complications. The elastic modulus of metallic materials is significantly higher than that of cortical or trabecular bone, leading to stress shielding. This phenomenon promotes bone resorption due to reduced physiological loading, ultimately weakening the surrounding bone and increasing the risk of implant loosening [13,14,15].

Metallic implants are also susceptible to bacterial colonization, particularly in dental applications where microbial exposure is continuous. Surface wear, microgaps at the implant–abutment junction, and corrosion promote environments where bacteria can adhere and form biofilms, which are difficult to eradicate. These challenges have prompted a shift toward the development of biodegradable and bioactive materials that can better mimic the natural healing process [16,17,18,19]. Among these materials, biodegradable polymers have attracted considerable attention due to their ability to degrade in vivo while supporting tissue regeneration and controlled drug delivery. Early work on synthetic biodegradable polymers established their potential for orthopedic and biomedical applications, particularly in reducing the need for secondary surgical removal [20]. Composite systems combining polymers with bioactive ceramics further improved osteoconductivity and mechanical performance, marking a significant step toward functional biomaterials for bone repair [21].

Among these candidates, poly(lactic-co-glycolic acid) (PLGA) has emerged as one of the most promising biomaterials due to its favorable combination of biocompatibility, tunable degradation kinetics, and regulatory approval for clinical use. Extensive research has demonstrated its versatility in drug delivery systems, nanoparticles, and scaffold fabrication [22,23]. Over the past decade, increasing attention has been directed toward its application in implantology, including its use in guided bone regeneration membranes, implant coatings, and multifunctional scaffolds.

Several reviews have previously examined PLGA-based systems; however, most have focused on general drug delivery applications, specific fabrication techniques, or broad tissue engineering strategies [23,24,25]. Recent advances—including nanocomposite design, additive manufacturing, and stimuli-responsive systems—have not been systematically integrated within an implantology-specific framework [24,25,26].

This review was conducted through a structured literature analysis using databases including Scopus, Web of Science, PubMed, and ScienceDirect, covering publications from 2010 to 2025, with particular emphasis on advances reported between 2020 and 2025. Therefore, this review provides a comprehensive and application-oriented evaluation of PLGA-based systems, specifically within the context of implantology.

Unlike prior studies, it establishes a quantitative and application-specific framework, directly correlating LA:GA ratio, molecular weight, and processing methods with implant-relevant biological outcomes, including osseointegration, degradation kinetics, and host response. Furthermore, recent clinical and translational developments (2024–2025), including 3D-printed guided bone regeneration (GBR) membranes and multifunctional coatings, are critically integrated to bridge the gap between material design and clinical application. By correlating physicochemical properties with biological performance and emerging clinical evidence, this review aims to provide a rational foundation for the design and translation of next-generation PLGA-based implantable biomaterials.

## 2. Physicochemical Properties of PLGA

**Figure 1:**
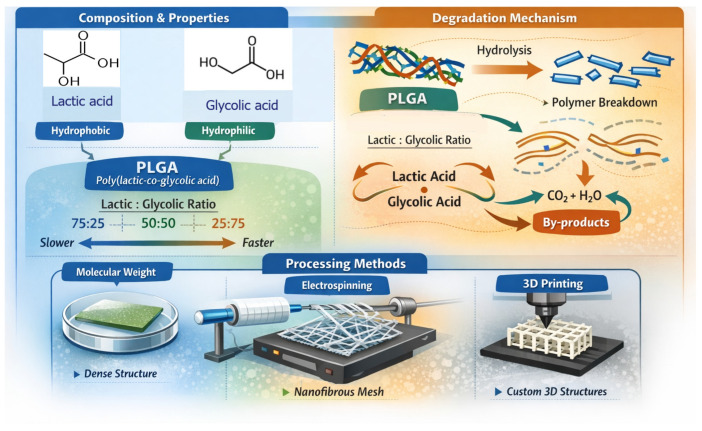
Schematic representation of PLGA physicochemical properties and their influence on degradation behavior and processing methods.

PLGA is a biodegradable and biocompatible copolymer widely used in drug delivery, tissue engineering, and implantable devices due to its tunable physicochemical properties [22,23,25,27]. Its performance is primarily governed by copolymer composition (LA:GA ratio), molecular weight, and processing conditions, which together determine degradation behavior and mechanical integrity (Figure 1) [23,25,28,29].

Molecular weight plays a central role in determining mechanical integrity, with higher molecular weight PLGA exhibiting greater structural stability, while lower molecular weight systems are more suitable for faster-resorbing applications [23,25,28,29,30,31]. Processing techniques further influence material performance by controlling microstructure and surface properties. Solvent casting produces dense films suitable for coatings, electrospinning generates highly porous fibrous matrices that enhance cell attachment, and 3D printing enables precise control over scaffold architecture and geometry [32,33,34].

These parameters highlight the importance of application-specific polymer design, as variations in composition and processing significantly influence in vivo performance (Table 1).

## 3. Biocompatibility and Biodegradation Behavior

PLGA is extensively used in biomedical applications due to its well-established biocompatibility and predictable biodegradation profile [35,36]. In vitro studies consistently demonstrate minimal cytotoxicity and normal cellular morphology, while in vivo studies confirm gradual resorption accompanied by tissue integration and a physiological inflammatory response comparable to natural remodeling processes [25,37].

The degradation products of PLGA—lactic acid and glycolic acid—are metabolized via the tricarboxylic acid cycle, minimizing systemic toxicity. However, degradation behavior is highly dependent on material design and device architecture, making it a critical parameter in implant performance [38,39].

### 3.1. Biodegradation Mechanisms

PLGA degrades predominantly through bulk hydrolysis of ester bonds, leading to progressive chain scission throughout the polymer matrix [40]. This process involves water penetration, reduction in molecular weight, and the eventual formation of soluble oligomers and monomers [38,41]. Unlike surface-eroding polymers, PLGA undergoes bulk erosion, resulting in gradual loss of mechanical integrity over time [38,41].

**Figure 2:**
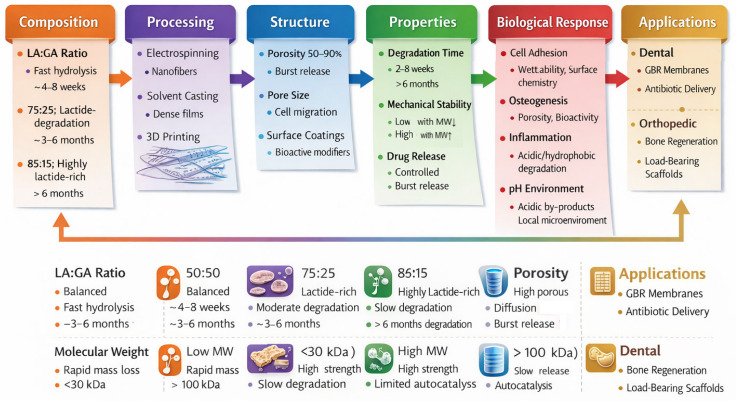
Quantitative integrated framework for PLGA-base implantology.

Degradation kinetics in PLGA systems are governed by a set of interrelated design parameters (Figure 2). The lactide:glycolide (LA:GA) ratio is the primary determinant of degradation rate, with PLGA 50:50 exhibiting the fastest hydrolysis (approximately 4–8 weeks), while increasing lactide content (e.g., 75:25 or 85:15) slows degradation due to greater hydrophobicity [23,42]. Molecular weight further influences degradation behavior, as high-molecular-weight PLGA (>100 kDa) retains structural integrity over longer periods, whereas low-molecular-weight systems (<30 kDa) degrade more rapidly and are better suited for drug delivery applications [43].

Porosity and scaffold architecture also play a critical role by regulating water diffusion. Highly porous structures (>70% porosity with interconnected pores > 100 µm) facilitate cell infiltration but accelerate degradation, whereas dense matrices restrict diffusion and promote internal accumulation of acidic degradation products. In larger or compact constructs, this leads to autocatalytic degradation, where internal regions degrade faster than the surface, resulting in heterogeneous structural breakdown [44,45].

To mitigate these effects, buffering strategies such as the incorporation of basic salts, hydroxyapatite (HA), or magnesium-based fillers are used to neutralize acidity and stabilize the microenvironment [23,39].

These factors collectively define a structure–property–degradation relationship, which must be tailored to match the functional lifetime required for specific implant applications.

### 3.2. In Vitro vs. In Vivo Degradation Behavior

Although in vitro studies provide controlled insight into hydrolytic degradation, they do not fully replicate the complexity of in vivo conditions, where enzymatic activity, fluid dynamics, and cellular interactions influence degradation behavior [31,41,46]. As a result, degradation rates and mechanisms may differ significantly between experimental models and clinical scenarios.

In dental applications such as guided bone regeneration, membranes must maintain functionality for approximately 4 weeks to 6 months while preserving mechanical stability [47,48]. For instance, PLGA bilayer membranes have demonstrated limited mass loss (<10% after 60 days) and suitable porosity for osteoblast infiltration, aligning with clinical requirements for bone regeneration [48].

### 3.3. Surface Modification to Improve Integration

Surface modification strategies for PLGA can be broadly classified into physicochemical activation (e.g., plasma treatment), bioactive coatings (e.g., calcium phosphates), and biomolecular functionalization (e.g., collagen or peptides). Rather than acting independently, these approaches modulate key surface parameters—wettability, roughness, surface energy, and biochemical signaling—which collectively determine protein adsorption and subsequent cell behavior. Compared to untreated PLGA, plasma-modified surfaces consistently show improved hydrophilicity and cell adhesion; however, excessive surface oxidation may accelerate degradation. Thus, an optimal balance between surface activation and structural stability is required. In contrast, bioceramic coatings such as hydroxyapatite provide osteoconductive cues, although they may alter degradation kinetics and mechanical integrity. Biomolecular modifications, including collagen incorporation, introduce specific ligand–receptor interactions, enabling more physiologically relevant cell responses. Furthermore, surface modification strategies differ in durability, with plasma treatments providing transient effects, while coatings offer more sustained functionality. Therefore, optimal implant performance requires multi-scale surface engineering, balancing initial cell attachment with long-term osteogenic signaling [49].

#### 3.3.1. Plasma Treatment

Exposure to oxygen or argon plasma introduces polar functional groups (e.g., –OH, –COOH) at PLGA surfaces without altering the bulk, significantly increasing surface hydrophilicity and protein affinity, which in turn improves cell attachment and proliferation (Table 2). Studies show that gas plasma treatment of PLGA surface leads to a significant decrease in the water contact angle, which translates into an enhanced cell binding affinity [50,51,52]. Furthermore, oxygen plasma treatment modifies the surface topography of the polymer membrane, increasing its roughness and thus improving its osteoinductive properties [53,54,55].

#### 3.3.2. Bioceramic Coatings

Calcium phosphate ceramics such as tricalcium phosphate and hydroxyapatite are biocompatible, naturally occurring minerals used for bone regeneration [59,60,61]. Hydroxyapatite is an inorganic component characterized by mechanical strength and hardness that is chemically and conformationally similar to both bone and teeth phosphate complexes [60,61,62]. However, these materials, hydroxyapatite in particular, do not degrade at a fast-enough rate to allow new bone growth [59]. By contrast, PLGA degrades at a predictable rate but has low osteoconductive properties [59]. Biomimetic HA coatings provide an osteoconductive interface that encourages bone-cell adhesion and mineralization when applied to PLGA scaffolds (Table 3) [59,60]. Hydroxyapatite particles increase the osteogenic properties of PLGA as they provide new sites for propagation and tissue attachment [51]. For instance, HA coatings on electrospun PLGA fibers enhance mesenchymal stem cell (MSC) spreading and osteogenic differentiation compared to unmodified PLGA. Moreover, hydroxyapatite has a drug loading capacity of 2 to 10 wt% which is significantly increased by the addition of PLGA. A prolonged release for up to 30 days was also observed [60]. Fu et al. successfully developed five types of functionally graded bilayer membranes containing different nHA/(PLGA + nHA) mass fractions. The mechanical properties of the membranes were not compromised with the incorporation of up to 30% nHA into PLGA. The study found that the addition of nHA could accelerate the biodegradation rate of the membranes in order to match the healing and regeneration process due to an increase in hydrophilicity. Furthermore, a high nHA content increased osteoblast attachment, proliferation and differentiation in vitro [63].

Beta-tricalcium phosphate (β-TCP) has a denser porous structure resulting in better bone replacement results when compared to hydroxyapatite. However, after implantation, it partially converts to HA, exhibiting an unpredictable biodegradation profile. Furthermore, β-TCP shows inferior mechanical properties [64]. In an exploratory study, Zhang et al. used an absorbable β-TCP/PLGA (30% β-TCP and 70% PLGA) spacer implantation in patients with medial compartmental knee osteoarthritis who underwent open-wedge high tibial osteotomy after proximal fibular osteotomy. Rejection and malabsorption observed in patients who underwent allogeneic bone grafting were eliminated when using the absorbable spacer due to its biocompatibility and biodegradability. Furthermore, the spacer accelerated the healing process and supported the osteotomy gap. However, a major drawback of the spacer was that full weightbearing was recommended only when the osteotomy gap was well-healed, at approximately 3 months postoperatively [65].

**Table 3:** Effects determined by calcium phosphate ceramics functionalization.

Scaffold	Method	Effect	Study
PLGA/nHA composite bone graft (surface-exposed nHA)	Gas foaming/particle leaching (GF/PL)	Enhanced osteogenic properties	[59]
Bilayer PLGA membranes (dense PLGA layer + electrospun PLGA/HA–β-TCP layer)	Phase inversion and electrospinning	Promotes osteoblast adhesion, proliferation, and migration; dense layer (~4.2 µm pores) prevents fibroblast infiltration; <10% mass loss after 60 days	[48]
PLGA and PLGA/HA scaffolds	-	Improved osteoconductivity in vitro and enhanced bone differentiation in vivo	[66]
PLGA/fish collagen/nHA fibrous membrane	Electrospinning	Improved cytocompatibility; enhanced osteogenic differentiation; supports new bone formation without systemic toxicity	[67]
HA loaded PLGA	PLGA grafting	Increased stability (up to 9 weeks); supports preosteoblast viability and differentiation	[68]
PLGA-coated calcium phosphate (CaP) on titanium substrates	Micro-arc oxidation method and dip coating	Improved mechanical stability and corrosion resistance in physiological conditions.	[69]
PLGA/nHA functionally graded bilayer membranes with different structures and surfaces	Phase inversion and electrospinning	Mechanical properties similar to commercial membranes; increased osteoblastic activity	[63]
PLGA/HA composites	Melt grafting with transesterification	Increased tensile strength (≈2× higher than blended systems)	[70]
PCL/PLGA/β-TCP membranes	3D printing	Comparable biocompatibility and bone regeneration to collagen membranes; improved mechanical stability in wet conditions	[71]
Porous PLGA/nHA scaffolds	Solvent evaporation and crosslinking	Cytocompatible structure with ~200 µm pores and interconnected porosity	[72]
nHA/PLGA/gelatin/platelet-rich fibrin (PRF) scaffold	Solvent casting/particulate leaching	Promotes osteoblast adhesion and proliferation	[73]
PLGA/HA composite scaffolds	Double emulsion and melt-sintering	Enhances osteoinduction and mineral deposition	[74]

Despite their osteoconductive advantages, calcium phosphate-based modifications introduce important trade-offs. Increased ceramic content enhances bioactivity but may lead to brittleness, reduced processability, and altered degradation kinetics. Additionally, discrepancies between in vitro and in vivo resorption rates remain a major limitation, particularly for β-TCP, which may undergo unpredictable phase transformation into hydroxyapatite. Consequently, the design of PLGA–bioceramic composites must carefully balance mechanical integrity, degradation behavior, and biological performance, depending on the intended clinical application (Table 3).

#### 3.3.3. Collagen Functionalization

Collagen type I represents a major component of the natural bone extracellular matrix and it is extensively used in tissue engineering. Under physiological conditions, collagen molecules self-assemble into fibrils with high mechanical strength and low immunogenicity. This allows collagen-based systems to form crosslinked hydrogels at 37 °C [75,76]. It also plays an important role in early cell attachment, migration and spreading of bone cells [76,77]. Collagen immobilization either through chemical coupling or incorporation into blended scaffolds increases integrin-mediated cell adhesion, mimicking extracellular matrix cues for improved tissue regeneration [78,79]. Multilayered 3D scaffolds containing sequential arrangements of microfibrous PLGA meshes with micro/nano mixed fibrous meshes of PLGA and collagen with and without glutamate-functionalized nHA hydroxyapatite nanorods were successfully fabricated using the dual extrusion electrospinning technique. Both collagen and hydroxyapatite nanorods increased the bioactivity of the scaffolds in terms of adhesion, proliferation, and osteogenic differentiation of MC3T3-E1 cells [76]. Moreover, a 3D composite scaffold based on type I collagen and transforming growth factor beta 1 (TGF-β1) encapsulated PLGA nanoparticles developed by Banche-Niclot et al. was able to mimic TGF-β1 distribution in the natural bone extracellular matrix [75].

Surface engineering strategies not only promote early biological integration but also modulate degradation profiles by altering wettability and protein interactions, enabling more predictable device performance in complex physiological environments [80,81].

Despite the diversity of surface modification techniques, key governing parameters include surface roughness, wettability, chemical functionality, and protein adsorption capacity. Plasma treatments primarily enhance hydrophilicity and initial cell adhesion, while bioceramic coatings improve osteoconductivity and long-term integration. In contrast, polymer–protein hybrid systems such as collagen-functionalized PLGA better mimic extracellular matrix signaling. The selection of the surface modification strategy should therefore be guided by the desired balance between early cell attachment and long-term tissue integration [55,59,64,75].

## 4. PLGA in Implantology

### 4.1. Drug Delivery Systems for Implants

Bone-tissue engineering ensures regeneration through 3D scaffolds that yield a controlled delivery of osteoinductive agents [82,83]. Scaffolds provide structural support while aiding bone mineralization and promoting cell adhesion [82,83,84,85]. Biodegradable drug delivery systems have the major benefit of not requiring secondary surgical removal from the body after implantation [86]. Biodegradable implants should be gradually overgrown with natural tissue while maintaining their shape and mechanical performance [87,88]. Scaffold morphology, composition, and geometry strongly influence both degradation and drug release. Increased porosity enhances diffusion and drug penetration, whereas thicker or denser structures delay degradation and reduce burst release. Composite systems incorporating ceramic fillers may further modify degradation behavior and release kinetics [51,86,87,88,89].

Polymeric biomaterials represent a good alternative for the fabrication of resorbable implants due to their tunable physicochemical properties and compatibility with various manufacturing methods. Moreover, PLGA can be manufactured through a number of different methods such as solvent-based processing, melt processing, and 3D printing into customable complex shapes [86]. PLGA-based drug delivery systems represent a cornerstone of modern implant design, enabling localized and sustained release of therapeutic agents directly at the implantation site (Table 4). This approach minimizes systemic exposure while maintaining effective local concentrations, thereby improving both efficacy and safety. Therapeutic agents are typically incorporated via physical strategies such as adsorption or encapsulation in order to preserve molecular integrity [83]. While PLGA implants enable localized, prolonged drug release directly at the bone site, PLGA microparticles can also be administered systemically to achieve targeted delivery in oncology treatments [86,90].

Drug release from PLGA matrices typically follows a multiphasic profile, characterized by an initial burst release of surface-associated molecules, followed by a diffusion-controlled phase and, ultimately, a degradation-driven release as the polymer matrix undergoes hydrolysis [86]. While this release behavior can be advantageous for rapid infection control, particularly in antibiotic-loaded systems, uncontrolled burst release remains a critical limitation that may compromise therapeutic precision. Strategies such as modulation of scaffold architecture, incorporation of ceramic fillers, and optimization of polymer composition have been employed to mitigate this effect and achieve more predictable release kinetics [86,90,91].

**Table 4:** Drug delivery systems for implants.

Drug Class	Drug	Preparation Method	Implant Characteristics	Reference
Anti-inflammatory agents	Ibuprofen	Hot melt extrusion, 3D printing	Pronounced initial burst release followed by tunable sustained release; release kinetics dependent on implant geometry and infill density; thermal processing influences drug loading	[89,92,93] (general implant)
	Ketoprofen	3D printing, double emulsion, sintering	Burst release influenced by printing parameters; dual delivery system (ketoprofen + bone morphogenetic protein-2 (BMP-2)) enables rapid anti-inflammatory action with prolonged protein release (~70 days)	[94,95] (orthopedic)
	Meloxicam	Airbrush spray coating	Sustained release (>30 days); improved corrosion resistance; enhanced cell adhesion and proliferation	[96] (orthopedic)
Anesthetics	Bupivacaine	Hot molding, solvent evaporation	Initial burst release within 24 h followed by sustained release	[97,98] (general implant/dental)
Antibiotics—β-Lactam antibiotic	Amoxicillin	Electrospray deposition	Controlled release (~2 weeks); effective against *Staphylococcus aureus* (*S. aureus*) and *Staphylococcus epidermidis* (*S. epidermidis*); supports osteoblast attachment	[99,100] (dental)
	Nafcillin	Single emulsion/solvent evaporation	Controlled release; strong antibacterial activity against *S. aureus*	[101] (orthopedic)
Antibiotics—aminoglycosides	Amikacin	3D Printing and Drop Casting Technique	Sustained release with prolonged antibacterial activity against *S. epidermidis* and *Methicillin-resistant Staphylococcus aureus* (MRSA)	[102] (orthopedic)
	Gentamicin	Airbrush spray, double emulsion, hot melt extrusion,	Sustained release (up to 6 weeks); inhibits biofilm formation; broad-spectrum antibacterial activity (*S. aureus*, *S. epidermidis*, *Pseudomonas aeruginosa*); enhances cell adhesion	[103,104,105,106,107] (both)
Antibiotics— glycopeptides	Vancomycin (VAN)	Hot-compression, 3D printing, emulsion polymerization	Gradual in vitro release; sustained in vivo release (>6 weeks); promotes new bone formation	[108,109,110,111] (orthopedic)
Antibiotics—fluoroquinolones	Ciprofloxacin (CIP)	Reversed phase separation/coacervation process, double emulsion technique	High loading efficiency; prolonged release (up to 4 months); effective against *Escherichia coli* (*E. coli*), *S. aureus*, MRSA; supports bone formation in vivo	[112,113,114,115,116] (both)
	Enoxacin	-	Sustained release; antibacterial activity against *S. aureus*, *S. epidermidis*; inhibits osteoclast formation	[117] (orthopedic)
	Moxifloxacin	Foam replica technique	High drug loading; prolonged release; antibacterial efficacy against *E. coli* and *S. aureus*	[118,119,120] (both/dental)
	Ofloxacin	Double emulsion	High loading efficiency; extended release profile	[121] (orthopedic)
Antibiotics—macrolides	Clarithromycin	-	Sustained release (~4 weeks); enhances bone formation	[122] (orthopedic)
	Clindamycin	Double emulsion solvent evaporation	Sustained release (>12 weeks); promotes bone regeneration	[123] (dental)
	Rifampicin (RIF)	Nanoprecipitation, solvent casting	Sustained release (~21 days); broad-spectrum antibacterial activity; low cytotoxicity	[119,124,125] (orthopedic)
Antibiotics— oxazolidinones	Linezolid	3D printing	Biphasic release; sustained antibacterial effect against MRSA (≈1 month); enhances tissue integration and osteogenesis	[126,127] (orthopedic)
Antibiotics—tetracyclines	Doxycycline	Electrospinning	Antibacterial activity; supports bone repair	[128,129] (both)
	Minocycline	Electrospinning	Sustained release (~40 days); promotes osteoblast proliferation	[130] (dental)
Bisphosphonates	Alendronate sodium (ALN)	Double emulsion, chemical bonding	Sustained release; enhances osteoblast activity and implant stability; reduces bone resorption and inflammation	[128,131,132,133,134] (both)
	Risedronate	Double emulsion,	Sustained release; high loading efficiency; improves bone microarchitecture	[135,136,137] (orthopedic)
	Zoledronic acid (ZOL)	Reverse microemulsion, melt extrusion 3D printing	Initial burst followed by sustained release; increases bone mineral density and peri-implant bone formation	[138,139,140,141,142] (both)
Chemotherapy	Cisplatin	Double emulsion, 3D printing	Controlled release (~21 days); improved anti-tumor efficacy with reduced systemic toxicity	[143,144] (orthopedic)
	Doxorubicin	-	Inhibits tumor cell growth while maintaining osteoblast viability	[145,146] (orthopedic)
Growth factors	BMP-2	3D printing, emulsion polymerization	Sustained release; promotes osteogenic differentiation and new bone formation	[109,110,142,147,148] (both)
	Vascular endothelial growth factor (VEGF)	-	Sustained localized release; enhances cell proliferation and attachment	[149,150] (both)
Statins	Simvastatin	Double emulsion, foam replica method	Controlled release; promotes vascularization and bone regeneration	[151,152,153] (both)
	Fluvastatin	-	Sustained release; supports bone formation in GBR applications	[154,155] (dental)

Bone defects are among the most common clinical conditions and may arise from diseases such as osteoarthritis, osteomyelitis, and osteoporosis, as well as from trauma, post-traumatic complications, tumors, and skeletal abnormalities. These conditions can lead to chronic pain, deformity, and impaired function [131,132,133,134,135,136,137,138,139,140,141,142].

Osteoarthritis can lead to severe joint damage that may require replacement surgery. A common problem associated with replacement surgery is insufficient osseointegration, which can result in loosening of the bone implant. Bisphosphonates are anti-resorptive drugs used to treat osteoporosis. Incorporating bisphosphonates into implants improves osseointegration by increasing the number of osteoblasts while decreasing osteoclast activity [131,132,133,134,135,136,137,138,139,140,141,142]. Short-term controlled local release of alendronate from PLGA microspheres has been reported to increase BMP-2 protein levels in regenerated bone and promote de novo bone formation. This effect was accompanied by improved mechanical properties, rather than a reduction in bone resorption within the bone defect model. Thus, showing that short-term release of ALN at submicromolar concentrations can enhance bone regeneration and improve the quality of newly formed bone [133]. By contrast, dopamine-anchored alendronate-functionalized scaffolds displayed a sustained local release of alendronate, resulting in a dual effect: suppression of osteoclast-mediated bone resorption and enhancement of new bone formation. Additionally, the incorporation of hydroxyapatite and magnesium oxide in the scaffold improved the local microenvironment by buffering acidic degradation products, which is a limitation often observed in PLGA-based systems [131]. Gou et al. used PLGA as a drug delivery system for ZOL. In this study, the controlled release of medium and high doses of ZOL in treated rats resulted in significant new bone formation, particularly near the distal femoral epiphyseal plate [140]. PLGA scaffolds loaded with recombinant human bone morphogenetic protein-2 (rhBMP-2), either alone or in combination with anti-resorptive agents such as ZOL, ZOL bound to hydroxyapatite, or an inhibitor of nuclear factor kappa-B kinase (IKK inhibitor, PS-1145) were implanted into critical-sized femoral defects in rats and compared with rhBMP-2 delivered via collagen by Yu et al. The addition of ZOL/HA restored bone volume to levels comparable to collagen and improved bone mineral density. Incorporating anti-resorptive agents significantly enhanced bone formation within PLGA scaffolds. Cellular studies also confirmed good cell infiltration and proliferation within the scaffold [142].

Statins are a class of drugs used to reduce cholesterol levels. However, studies have shown that statins enhance bone regeneration by stimulating osteoblast activity and reducing bone resorption. Furthermore, the controlled release of simvastatin from microspheres promotes neovascularization and supports cell proliferation within transplanted grafts [151,152,153,154,155].

Inflammation, inadequate osseointegration, and biofilm formation may cause implant failure in orthopedic surgery. Implant-associated infections remain a significant challenge, as implant surfaces provide a favorable substrate for bacterial adhesion and biofilm formation. Incorporating antibiotics into bone scaffolds offers a promising solution, enabling high local antibiotic concentrations without systemic toxicity. Including vancomycin into PLGA delivery systems resulted in an initial burst release of the antibiotic for the first two days followed by sustained release. Lin et al. combined PLGA with vancomycin and lithium chloride to fabricate biodegradable drug delivery beads for the treatment of bone infections. The system exhibited an initial burst release of vancomycin within the first 48 h, effectively eliminating bacteria at the surgical site, followed by a sustained release that remained above the therapeutic threshold for up to 42 days. This prolonged release was attributed to the gradual diffusion of the drug from the bead matrix. The antibacterial efficacy of vancomycin was further enhanced by the presence of lithium. Additionally, lithium release promoted osteogenic differentiation of MSCs [108]. Another PLGA-based drug delivery system containing both vancomycin and rhBMP-2 demonstrated an initial rapid release of vancomycin within the first 2 days to control infection, followed by sustained release of rhBMP-2 for approximately 12 days to promote bone formation. Both in vitro and in vivo results confirmed its biocompatibility and effectiveness. This sequential delivery approach shows strong potential as a coating for dental implants to improve surface properties and enhance osseointegration after surgery [109]. PLGA-based delivery systems loaded with ciprofloxacin exhibited sustained release and antibacterial efficacy against *E. coli*, *S. aureus* and *MRSA* in vitro. PLGA-based cylindrical pellets incorporating CIP and bioactive glass microspheres obtained by Mäkinen et al. represented a promising approach for combined infection control and bone regeneration. The system provided sustained release of ciprofloxacin at therapeutic levels for up to three months, while maintaining its antimicrobial effectiveness against multiple bacterial strains. In vivo results further confirmed high local antibiotic concentrations with minimal systemic exposure, highlighting the advantage of localized drug delivery. Additionally, new bone formation was observed within three months through micro-CT analysis [114]. Furthermore, different biodegradable polymers including PLGA were used to create CIP-loaded coatings on a titanium-based alloy, designed for short-term therapeutic applications. The results showed that thin, well-adhered biodegradable polymer coatings exhibited effective bactericidal activity against *E. coli* [115]. PLGA delivery systems loaded with ciprofloxacin displayed a sustained drug release as well as antibacterial efficacy against *S. aureus*, *S. epidermidis*, *E. coli* and *Acinetobacter baumannii* (*A. baumannii*) [124,125]. RIF-loaded nanoparticles were obtained using the nanoprecipitation method. When incorporated into cancellous allogenic bone grafts, the nanoparticles displayed controlled local drug delivery, characterized by a low initial burst followed by sustained release over several days. This helps prevent resistant biofilm formation and supports the treatment of infections in musculoskeletal injuries [124]. Furthermore, PLGA-RIF composite membranes obtained by Singhal et al. exhibited controlled drug release over 21 days in vitro. Additionally, the increase in inhibition zones over time against several strains, namely *S. aureus*, *S. epidermidis*, *E. coli*, and *A. baumannii*, highlights their ability to sustain effective RIF concentrations and inhibit bacterial growth [125].

Localized delivery of anti-inflammatory agents represents another promising approach to mitigate complications by reducing inflammation, enhancing osseointegration, and limiting biofilm development [96,100,106,117]. Meloxicam–PLGA-coated implants displayed greater resistance to pitting corrosion, along with enhanced cell adhesion and proliferation compared to uncoated implants. Furthermore, cytotoxicity assays showed that all coatings were biocompatible and suitable for in vivo use. Implants also exhibited significantly reduced bacterial adhesion and biofilm formation for both *S. aureus* and *Staphylococcus pseudintermedius*. Ultimately, meloxicam–PLGA-coated implants showed promise in improving implant integration and may help reduce the risk of implant rejection in orthopedic surgery patients [96].

Emerging approaches increasingly incorporate bioactive nanoparticles and natural compounds to enhance functionality. For example, biosynthesized selenium nanoparticles have demonstrated significant antimicrobial and cytotoxic activity, suggesting their potential as adjuncts in PLGA-based coatings or scaffolds to further inhibit biofilm formation and improve implant-associated infection control [156]. Furthermore, plant-derived bioactive compounds have gained attention as eco-friendly alternatives for enhancing implant functionality. Extracts rich in phenolic compounds, such as those derived from Glycyrrhiza glabra roots, have demonstrated significant antimicrobial activity [157]. These natural agents could be incorporated into PLGA-based coatings to further inhibit bacterial adhesion and biofilm formation on both metallic and biodegradable implant surfaces, offering a sustainable and biologically active modification approach. These hybrid systems reflect a shift toward multifunctional platforms that combine structural support with targeted biological activity.

The range of therapeutic agents incorporated into PLGA-based implant systems highlights the versatility of this platform; however, clear trends emerge when comparing drug classes, fabrication methods, and resulting release profiles. Across studies, most systems exhibit an initial burst release followed by a sustained phase, although the magnitude of this burst is highly dependent on the preparation technique. For example, surface-based approaches such as coating or electrospinning tend to produce faster release due to increased surface area, whereas encapsulation methods such as double emulsion or solvent evaporation enable more prolonged and controlled delivery, in some cases extending to several weeks or months. Advanced fabrication strategies, including 3D printing and hot melt extrusion, further allow the modulation of release kinetics through structural parameters such as porosity, infill density, and implant geometry. A notable distinction is observed between drug classes in terms of therapeutic objectives and release requirements. Antibiotics dominate the field, reflecting the clinical importance of infection prevention in implantology, and are typically designed for sustained antibacterial activity over several weeks. In contrast, anti-inflammatory agents and anesthetics often rely on a more pronounced initial burst to address acute postoperative responses. Meanwhile, osteoactive compounds such as bisphosphonates, growth factors, and statins are engineered for prolonged release to support bone regeneration and remodeling. Importantly, several studies demonstrate the advantage of dual or sequential delivery systems, where an early release of antimicrobial or anti-inflammatory agents is combined with sustained delivery of osteogenic factors, mimicking the natural healing cascade (Table 4).

Beyond individual examples, a set of overarching design principles can be identified for PLGA-based implant drug delivery systems. The polymer composition, particularly the LA:GA ratio, plays a central role in regulating degradation and thus controlling release kinetics. Structural features such as porosity and overall geometry influence diffusion behavior and the magnitude of any initial burst release. In addition, the method used to incorporate the drug—whether through encapsulation or surface adsorption—directly affects the release profile. The use of composite systems, for example with HA or magnesium oxide (MgO), can further enhance performance by buffering acidic degradation products and stabilizing drug release.

Despite these advances, direct comparison between systems remains challenging due to variability in experimental design, drug loading, and evaluation methods. This underscores a critical gap in the field: the need for standardized approaches to correlate fabrication techniques with release behavior and clinical outcomes. Overall, the data presented in Table 4 indicate a clear transition from simple drug carriers toward increasingly sophisticated, programmable delivery platforms tailored to the temporal and biological demands of implant healing.

### 4.2. Orthopedic Implantology Applications

#### 4.2.1. Load-Bearing Scaffolds for Bone Defect Repair

An ideal bone graft should replace a bone defect that resulted from trauma or a disease with a composite that allows and stimulates bone-cell growth. Autografts are considered the standard for bone repair but they require a second surgical site in order to remove the bone. Researchers have successfully developed biomaterials for the development of bone grafts. Orthopedic applications impose significantly different requirements compared to dental systems, particularly in terms of mechanical strength and load-bearing capacity. PLGA-based scaffolds are widely used for treating critical-sized bone defects, often in combination with bioactive ceramics. Therefore, they should promote bone-cell propagation on the surface of the graft and degrade in time for new bone to grow [57]. The development of three-dimensional scaffolds that are non-toxic and display good mechanical properties, biodegradability and porosity is essential in bone-tissue regeneration [72]. Furthermore, scaffolds must ensure bone-forming cell adhesion and growth [73]. Unlike dental GBR membranes, orthopedic scaffolds must provide temporary mechanical support while gradually transferring load to regenerating bone, minimizing stress shielding effects. This is typically achieved through composite systems (PLGA/nHA, PLGA/β-TCP) that enhance stiffness and osteoconductivity. However, increasing ceramic content introduces trade-offs, including brittleness and reduced processability. Additionally, the mismatch between scaffold degradation and bone healing rates remains a major limitation, particularly in large defects. There are several methods used for fabricating porous 3D scaffolds such as 3D printing, freeze-drying, bioprinting, lyophilization, phase separation and gas foaming, ensuring an enhanced regeneration process [72]. PLGA, in combination with nHA, produces a composite with high porosity (Table 2). Moreover, the pore size of the scaffold may be adjusted to better mimic the natural bone structure [73]. According to Klar et al., PLGA microsphere scaffolds support bone regeneration by promoting osteoconduction due to their unique geometric configuration even in the absence of osteoinductive factors [158]. Additionally, loading growth factors into artificial scaffolds improves osteogenic properties, as they play a key role in tissue remodeling. Currently, growth factors such as VEGF, basic fibroblast growth factor (bFGF) and rhBMP-2 are widely used to treat bone defects and spinal cord injuries [51,73]. Furthermore, PLGA scaffolds ensure the delivery of sensitive bioactive materials such as PRF for bone-tissue engineering [73].

Sokolova et al. obtained and characterized PLGA/nHA scaffolds and PLGA/nHA scaffolds incubated with DNA-functionalized calcium phosphate nanoparticles (Enhanced Green Fluorescent Protein (EGFP)). PLGA/nHA scaffolds showed high biocompatibility. Furthermore, they allowed the delivery of DNA plasmids into cells which lead to an expression of EGFP, making them a promising alternative in bone-tissue engineering [72]. Chemokine interleukin-8 and transferable decellularized matrix were added on exterior and interior surfaces of PLGA substrates resulting in a scaffold with osteoinductive properties. The scaffold increased chemotaxis, spreading, adhesion, osteogenic mineralization and differentiation of bone marrow mesenchymal stem cells in vitro and better bone repair ability than control groups in vivo [159]. However, a study conducted by Cushnie et al. determined that the addition of growth factors to PLGA/HA composite scaffolds does not further enhance scaffold osteoinductivity compared to PLGA/HA scaffolds [74].

#### 4.2.2. Coatings for Metallic Implants

Metallic implants such as stainless steel, titanium alloys and cobalt chrome alloys are widely used in orthopedic surgical procedures [160]. Titanium and its alloys remain the standard in orthopedics as they exhibit high biocompatibility, superior mechanical properties and resistance to corrosion compared to stainless steel and other metal alloys [160,161]. However, several drawbacks such as poor osseointegration and implant-associated infections are common complications causing pain and inflammation and ultimately leading to poor quality of life for patients [160,161].

PLGA-based coatings provide a multifunctional solution by combining corrosion protection, drug delivery, and surface bioactivation [161]. Furthermore, PLGA coatings incorporating antibiotics or nanoparticles have been shown to reduce bacterial adhesion, improve corrosion resistance, and enhance osteoblast proliferation. Titanium plates coated with PLGA or chitosan with meropenem displayed an increased antibacterial efficacy compared to meropenem without polymers. This was achieved through the combined inhibition of bacterial adhesion, biofilm formation, and bacterial proliferation [160]. Titanium–copper alloy implants coated with nanosilver/PLGA determined excellent bone induction both in vivo and in vitro as well as antibacterial action against both Gram-negative *E. coli* and Gram-positive *S. aureus* due to the release of silver and copper ions [85]. Prosolov et al. developed PLGA/CaP/Ti scaffolds containing different PLGA concentrations, namely 5%, 8% and 11%, as orthopedic implants that displayed high corrosion resistance and better mechanical properties for higher PLGA concentrations [69]. Another method for solving implant infection-related problems is by including antibiotics in titanium scaffolds [86,113,162,163,164,165]. Coating titanium dioxide nanotubes with vancomycin and PLGA increased titanium scaffold biocompatibility, exhibiting lower toxicity and improved proliferation of bone marrow stem cells [162].

Metallic implants may require a secondary surgery for implant removal due to complications such as pain and inflammation, risk of infection, interference in tissue growth, corrosion-related allergic reactions, implant loosening and breakage [50,151,166]. In recent years, Mg- and Zn-based implants have been developed for fracture fixation in younger patients where another secondary surgery may possess a high risk [86,167,168]. Mg-based alloys exhibit suitable mechanical and physical properties allowing the fabrication of biodegradable metallic implants. However, studies showed that magnesium-based implants may display structural and mechanical problems due to elevated degradation rates [86,169]. For Mg alloys, PLGA coatings play an additional role in controlling degradation rate, preventing rapid corrosion and hydrogen gas formation. PLGA particles containing 2-mercaptobenzimidazole and curcumin were successfully obtained and used to coat AZ31 magnesium alloy implants, resulting in a reduced corrosion rate of the AZ31 alloy in testing environments and reduced bacterial growth, thereby lowering infection risks associated with implants. Furthermore, the PLGA coating demonstrated high cell viability, with approximately 81% of cells remaining viable after 24 h [168]. A different study confirms that surface modifications of AZ31 alloy with polylactic acid and PLGA layers improved the resistance to corrosion in simulated body fluid solution. Moreover, they promote osseointegration and cell proliferation while being able to suppress the long-term release of Al^3+^ ions into the simulated body fluid [169].

### 4.3. Dental Implantology Applications

#### Guided Bone Regeneration Membranes

Guided bone regeneration is a well-established clinical procedure that employs a barrier membrane to isolate the bone defect from the surrounding soft connective tissues and prevent the infiltration of rapidly proliferating fibroblasts. This selective exclusion creates a protected environment that allows sufficient time for osteogenic cells to repopulate the defect site, thereby promoting effective bone regeneration [48,63,170,171]. As such, GBR represents one of the most established clinical applications of PLGA in dental implantology.

Clinically, GBR is widely used to treat bone deficiencies arising from trauma, tumor resection, periodontitis, or prolonged tooth loss. It is currently recommended to use GBR technology before or during dental implantation for better implant success rate [63,171,172,173,174].

Membranes used for periodontal guided bone regeneration should comply with several key requirements, including biocompatibility, the ability to maintain space, effective cell occlusiveness, integration with host tissues, and ease of clinical handling. GBR membranes are divided into two groups: non-resorbable membrane and bioabsorbable. Clinically, GBR is widely used to treat bone deficiencies arising from trauma, tumor resection, periodontitis, or prolonged tooth loss. Consequently, there has been a clear shift toward bioresorbable materials such as collagen and synthetic polymers, particularly PLGA [63,171].

Despite their advantages, resorbable materials present significant limitations such as reduced mechanical strength and unpredictable resorption time which may be insufficient to support bone regeneration before the membrane degrades. PLGA offers the advantage of a tunable degradation rate, which can be tailored to align with the time needed for bone regeneration. This is achieved by adjusting the ratio of lactic acid to glycolic acid [171,172,173,174].

Recent advancements in PLGA-based GBR membranes have focused on improving both biological performance and mechanical stability. As summarized in Table 5, different material strategies have been explored to optimize these properties. For instance, bilayer PLGA membranes demonstrate minimal degradation (<10% mass loss over 60 days) while maintaining structural stability and supporting effective bone formation. Composite systems incorporating bioactive fillers such as nHA further enhance osteoblast activity and promote osteogenic differentiation. Similarly, hybrid formulations such as PCL/PLGA/β-TCP exhibit improved mechanical strength under wet conditions, addressing one of the key limitations of conventional resorbable membranes and resulting in enhanced bone-to-implant contact [48,63,71,171,172]. These findings highlight a clear trend toward multifunctional and composite membrane designs that balance degradation, mechanical integrity, and biological activity. However, trade-offs remain, as increasing inorganic filler content may reduce membrane flexibility and negatively impact clinical handling. Overall, current evidence suggests that the next generation of PLGA-based GBR membranes is evolving from passive barrier systems toward actively engineered platforms capable of supporting both structural and biological requirements in implant dentistry.

### 4.4. Dental vs. Orthopedic Applications

The comparison between dental and orthopedic applications highlights that PLGA-based systems must be tailored to fundamentally different clinical and mechanical environments (Table 6). In dental implantology, mechanical demands are typically low to moderate, as most applications involve non-load-bearing systems such as GBR membranes or coatings. Consequently, shorter degradation times of the order of weeks to months are often preferred, aligning with the need for temporary barrier function and localized infection control in a highly moist and bacteria-rich oral environment. In this context, PLGA is typically processed into thin membranes or coatings, frequently combined with antimicrobial agents to prevent peri-implant infections. The primary challenge, therefore, lies in maintaining material stability and functional integrity under constant exposure to saliva and fluctuating pH conditions. In contrast, orthopedic applications impose significantly higher mechanical demands, particularly in load-bearing regions where implants must provide structural support over extended periods. This necessitates longer degradation profiles, often spanning months to years, to ensure sustained mechanical integrity during tissue regeneration. PLGA in these settings is commonly engineered into three-dimensional scaffolds or composite systems, frequently reinforced with bioactive ceramics to enhance strength and osteoconductivity. Drug delivery strategies also differ, shifting from predominantly antimicrobial roles in dentistry to more complex therapeutic goals, including osteogenic stimulation and modulation of bone resorption. Additionally, orthopedic interventions typically address larger defect volumes, further emphasizing the need for robust scaffold architecture and long-term performance. Together, these distinctions underscore that successful PLGA design cannot be generalized across fields, but must instead be application-specific, balancing degradation, mechanics, and biological function according to the clinical context.

## 5. Current Challenges and Limitations

PLGA-associated implant characteristics such as drug release profile, degradation kinetics, and mechanical performance may be adjusted through polymer composition and the addition of several excipients such as PEG, poloxamer or surfactants. Despite its versatility, PLGA-based systems face several limitations that hinder clinical translation. A major challenge is uncontrolled initial burst release, which can result in suboptimal therapeutic profiles or localized toxicity. Additionally, progressive loss of mechanical integrity during degradation limits performance in load-bearing applications [65,169]. PLGA/β-TCP composites did not produce better osseointegration compared to standard titanium-alloy implants in a minipig model [170]. Absorbable β-TCP/PLGA spacers in patients with medial compartmental knee osteoarthritis supported the healing process well but displayed mechanical instability under load which delayed weight bearing for 3 months [65].

Another critical issue is the accumulation of acidic degradation by-products (see Section 3). Strategies such as incorporation of buffering agents or bioactive fillers have been proposed to mitigate these effects. Natural sources such as Myrtus communis fruits, rich in bioactive phytochemicals with strong antioxidant properties, may provide a biologically compatible approach to mitigating oxidative stress and inflammation associated with PLGA degradation [175]. Furthermore, variability in polymer composition, fabrication methods, and environmental conditions contributes to inconsistent degradation behavior and drug release profiles, limiting reproducibility and clinical reliability. While advances in additive manufacturing and nanotechnology offer improved control over scaffold architecture, challenges related to scalability, cost, and regulatory approval remain substantial [165,176,177].

Importantly, although PLGA has a well-established safety profile, the translation of complex multifunctional systems into clinical practice is still limited. The lack of standardized in vitro–in vivo correlation models and long-term clinical data continues to represent a major barrier to regulatory approval and widespread adoption [51]. A thorough evaluation of long-term biocompatibility and interactions between the implant and the host immune system is necessary before clinical evaluation. Therefore, comprehensive studies demonstrating the safety and efficacy of drug delivery systems that promote osseointegration must be conducted to achieve regulatory approval.

## 6. Future Perspectives

PLGA scaffolds containing various fillers and drugs should ensure adequate mechanical support as well as deliver the active agent locally directly to a bone or dental site, thus improving the healing process. However, an increased percentage of the drug may be released during the initial burst release phase which does not ensure a controlled sustained release. The aim of research is to create a PLGA scaffold that will release the drug at a rate matching its specific application. Moreover, PLGA scaffolds must meet the characteristics of the tissue they replace. Furthermore, the degradation rate of biodegradable PLGA scaffolds should match the healing process of the implant site.

Newly developed technologies, such as 3D printing and electrospinning, are able to yield complex, patient-specific scaffolds with precise control over both pore architecture and geometry resulting in improved tissue osseointegration. Customized treatments with personalized drug delivery systems will ensure improved precision and effectiveness thus improving patient compliance. Proper regulatory and clinical advancements are required for the approval of these patient-specific innovations [87].

Smart drug delivery systems that respond to exogenous stimuli such as magnetic fields, ultrasound, light and temperature or to local microenvironments such as enzymatic activity or pH shifts allow on-demand therapeutic modulation [178,179,180,181,182,183]. These systems allow for localized drug delivery, reduced systemic toxicity, and potential for theranostics. However, there are a limited number of orthopedic applications that meet theranostic requirements such as adequate, tissue-specific accumulation and deep tissue penetration. Moreover, new developments in materials science show promise in the design of next-generation PLGA scaffolds customized for specialized applications [51,184].

The standardization of regulatory guidelines, advancements in analytical methodologies, as well as increased collaboration between regulatory authorities, researchers and manufacturers are essential in the approval process of PLGA-based products, enabling the development of next-generation medical devices [86].

## 7. Conclusions

PLGA-based systems have evolved from conventional biodegradable carriers into sophisticated multifunctional platforms capable of integrating structural support, controlled drug delivery, and bioactive signaling. Their tunable physicochemical properties, combined with compatibility with advanced fabrication techniques, position them as key materials in the development of next-generation implantable devices.

However, the successful translation of these systems requires a more precise alignment between material design and biological performance. Persistent challenges—including burst release, degradation-induced acidity, and mechanical limitations—highlight the need for improved design strategies and standardized evaluation models.

Future progress will depend on the integration of emerging technologies such as additive manufacturing, nanostructured composites, and stimuli-responsive systems, alongside robust clinical validation and regulatory harmonization. By bridging the gap between material science and clinical application, PLGA-based biomaterials hold significant potential to redefine the landscape of regenerative implantology.

## Figures and Tables

**Table 1:** Structure–property–application relationships in PLGA.

LA:GA Ratio	Molecular Weight	Degradation Time	Mechanical Strength	Application
50:50	Low	1–2 months	Low	Drug delivery
65:35	Medium	3–6 months	Moderate	GBR membranes
75:25	High	>6 months	High	Scaffolds
85:15	High	Slow	High	Load-bearing coatings

**Table 2:** Effects induced by plasma treatment-mediated surface modification of PLGA.

Plasma Treatment	PLGA Copolymer Ratio	Plasma Treatment Effect	Study
Oxygen plasma-treated parylene C with drug-loaded PLGA coating	PLGA 85:15	Enhanced corrosion resistance, antibacterial performance, and overall therapeutic efficacy	[56]
Argon plasma-treated PLGA, collagen, and PLGA–collagen films nano-hydroxyapatite (nHA) and poly(ethylene glycol) (PEG)	PLGA 82:18	Reduced protein release and increased scaffold stability; significant increase in surface hydrophilicity (contact angle reduced from ~70° to ~42°)	[52]
Oxygen plasma-functionalized PLGA membranes with silicon dioxide (SiO_2_) layers	PLGA membranes (polycondensation)	Improved bone regeneration and new bone formation in vivo (rabbit calvarial model)	[53]
Oxygen plasma-modified PLGA membranes with HA/SiO_2_/TiO_2_ nanoparticles	PLGA 75:25	Enhanced osteoblast morphology and cell viability; improved bioactivity compared to untreated membranes	[57]
Oxygen plasma-treated PLGA membrane films	PLGA 75:25	Induced surface roughness with porous morphology; highlights differential degradation behavior (glycolic units more susceptible than lactic units)	[58]

**Table 5:** Performance of PLGA-based GBR membranes.

Composition	Degradation	Mechanical Strength	Outcome	Study
PLGA bilayer	Minimal mass loss 10% loss (60 d)	Stable barrier integrity	Supports new bone formation and defect coverage	[48]
PLGA/nHA	Tunable via composition	Maintained structural stability	Enhances osteoblast adhesion and activity	[63]
PLGA + fish collagen + hydroxyapatite	Controlled degradation with improved bioactivity	Enhanced mechanical properties and flexibility	Promotes osteogenic differentiation and bone regeneration	[67]
PCL/PLGA/β-TCP	Controlled, prolonged degradation	High mechanical stability (wet conditions)	Improves bone–implant contact and regeneration	[71]
PLGA + fluvastatin	Gradual degradation (GBR-matched)	Moderate strength, suitable for membrane applications	Enhanced bone formation and regeneration	[154]
PLGA–HA bilayer (PLGA-grafted hyaluronic acid)	Controlled via bilayer architecture	Improved flexibility with maintained barrier integrity	Enhances periodontal regeneration and soft tissue integration	[172]

**Table 6:** Comparison of PLGA requirements in dental vs. orthopedic implantology.

Parameter	Dental Applications	Orthopedic Applications
Mechanical demand	Low–moderate	High (load-bearing)
Degradation time	Weeks–months	Months
Primary goal	Barrier function, infection control	Structural support, regeneration
Scaffold type	Membranes, coatings	3D scaffolds, composites
Drug delivery focus	Antimicrobial	Osteogenic + anti-resorptive
Key challenge	Moist environment stability	Mechanical integrity
Clinical scale	Small defects	Large defects

## Data Availability

No new data were created or analyzed in this study.

## Abbreviations

The following abbreviations are used in this manuscript:

PLGA	Poly(lactic-co-glycolic acid)
PLA	Polylactic acid
PCL	Polycaprolactone
nHA	Nano-hydroxyapatite
β-TCP	Beta-tricalcium phosphate
MSC	Mesenchymal stem cells
PRF	Platelet-rich fibrin
VEGF	Vascular endothelial growth factor
bFGF	Basic fibroblast growth factor
BMP-2	Bone morphogenetic protein-2
GBR	Guided bone regeneration
CaP	calcium phosphate
*E. coli*	Escherichia coli
*S. aureus*	Staphylococcus aureus
*S. epidermidis*	Staphylococcus epidermidis
MRSA	Methicillin-resistant Staphylococcus aureus
*A. baumannii*	Acinetobacter baumannii
CIP	Ciprofloxacin
RIF	Rifampicin
ALN	Alendronate sodium
ZOL	Zoledronic acid
